# COVID-19 and Hospital Financial Viability in the US

**DOI:** 10.1001/jamahealthforum.2022.1018

**Published:** 2022-05-13

**Authors:** Yang Wang, Ge Bai, Gerard Anderson

**Affiliations:** 1 Department of Health Policy and Management, Johns Hopkins Bloomberg School of Public Health, Baltimore, Maryland; 2 Johns Hopkins Carey Business School, Washington, DC; 3 International Health, Johns Hopkins Bloomberg School of Public Health, Baltimore, Maryland; 4 Johns Hopkins University School of Medicine, Baltimore, Maryland

## Abstract

**Importance:**

The COVID-19 pandemic has had a negative association with hospital operations. To help health care facilities and clinicians stay financially viable during the COVID-19 pandemic, Congress provided $175 billion in subsidies. It remains unclear how much financial losses hospitals incurred owing to operational disruptions during the COVID-19 pandemic and whether subsidies were sufficient to offset the financial losses.

**Objective:**

To assess changes in the operational financial performance and overall financial viability of hospitals during the COVID-19 pandemic.

**Design, Setting, and Participants:**

This cross-sectional study included 1378 US hospitals whose fiscal years began in January and 785 hospitals whose fiscal years began in July (all with continuous observations from 2016 through 2020). RAND Hospital Data, a compiled and processed version of Medicare Cost Reports, were used. The data were analyzed on March 12, 2022.

**Exposures:**

The operational disruptions experienced and relief funds received by US hospitals during the COVID-19 pandemic.

**Main Outcomes and Measures:**

A hospital’s annual operating margin, overall profit margin, and other nonoperating income as a share of total revenue from January 2016 to December 2020.

**Results:**

Among the 1378 hospitals with fiscal years beginning in January, the mean operating margin declined from –1.0% (95% CI,–1.9% to –0.1%) in 2019 to –7.4% (95% CI, –8.5% to –6.3%) in 2020. The mean share of other nonoperating income grew from 4.4% (95% CI, 4% to 4.7%) in 2019 to 10.3% (95% CI, 9.9% to 10.8%) in 2020. The mean overall profit in 2020 (6.7%; 95% CI, 5.4% to 8.1%) remained as stable as prior years. Government, rural, and smaller hospitals showed higher mean overall profit margins in 2020 than in 2019 (7.2% vs 3.7%, 7.5% vs 1.9%, and 6.7% vs 3.5%, respectively). These results remained consistent when hospitals whose fiscal years began in July were examined.

**Conclusions and Relevance:**

The results of this cross-sectional study suggest that although hospitals experienced a sizeable reduction in operating margins in 2020, their overall profit margins remained similar to those in prior years, suggesting that the COVID-19 relief fund effectively offset the financial losses for hospitals during the COVID-19 pandemic. Government, rural, and smaller hospitals, which were supported by some targeted fund allocations, generated higher overall profit margins during 2020 than in prior years.

## Key Points

**Question:**

How did the financial viability of US hospitals change during the COVID-19 pandemic?

**Findings:**

In this cross-sectional study of 2163 US hospitals, a sizeable reduction in the operating margins of US hospitals was found in 2020. However, their overall profit margins remained similar to those in prior years, and government, rural, and smaller hospitals generated higher overall profit margins during 2020 than in prior years.

**Meaning:**

The study results suggest that the COVID-19 relief fund effectively offset the operational financial losses of hospitals during the COVID-19 era, particularly for government, rural, and smaller hospitals, which are typically more financially vulnerable and have been supported by some targeted fund allocation.

## Teaser

This cross-sectional study examines changes in the operational financial performance and overall financial viability of US hospitals during the COVID-19 pandemic.

## Introduction

The COVID-19 pandemic has had a negative association with hospital operations, forcing them to restructure facilities to treat patients with COVID-19 and cancel elective procedures.^1,2^ To help hospitals and other health care facilities and clinicians remain financially solvent, the Coronavirus Aid, Relief, and Economic Security Act and Paycheck Protection Program and Health Care Enhancement Act provided $175 billion in subsidies.^1^ An important policy question is whether the subsidies were sufficient to offset the financial losses associated with operational disruptions. This study examined a full year of COVID-19 experience in 2020 using newly published 2020 Medicare Cost Reports on 1378 Medicare-certified general acute care hospitals whose fiscal years began on January 1, and compared it with their financial experience from January 2016 to December 2019. This approach avoids the measurement noise associated with variations in hospital fiscal year starting dates.

## Methods

This study used RAND Hospital Data (downloaded on February 2, 2022), a compiled and processed version of Medicare Cost Reports published by the US Centers for Medicare & Medicaid Services.^3^ Almost 40% of Medicare-certified general acute care hospitals in the US begin their fiscal years on January 1 (eTable 1 in the Supplement). These hospitals had more profitable operations and were less likely to be government hospitals and more likely to be in metropolitan areas than other hospitals (Table). Among these hospitals, only those with continuous observations from 2016 to 2020 and no reporting anomalies were examined to ensure a consistent study group (eTable 2 in the Supplement).^4^ The sample contained 1378 hospitals (6890 hospital-year observations).

**Table.** Comparison Between Hospitals With Their Fiscal Year Starting in January and Other Hospitals in 2018

Variable	Hospitals with fiscal years starting in January^a^	Other hospitals^b^	*P* value
Mean operating margin	–2.62	–5.92	<.01
Mean overall profit margin	3.32	3.05	.60
Mean share of other nonoperating income	3.84	5.08	<.01
Hospitals, No. (%)			
Nonprofit	977 (60)	1541 (59)	.67
For profit	407 (25)	411 (16)	<.01
Government	241 (15)	640 (25)	<.01
Metropolitan	1096 (67)	1465 (56)	<.01
Micropolitan	253 (16)	457 (18)	.08
Rural	275 (17)	668 (26)	<.01
No. of admissions	7264	7086	.58
No. of hospitals	1625	2592	NA

Abbreviation: NA, not applicable.

^a^ These hospitals include all hospitals whose fiscal years began on January 1, 2018, and ended on December 31, 2018, which did not overlap with the COVID-19 pandemic.

^b^ Other hospitals include all hospitals whose fiscal years started between October 1, 2017, and September 1, 2018 (not on January 1, 2018), which did not overlap with the COVID-19 pandemic.

Operating margin (net income from patient services divided by patient revenue, net of contractual allowances), which was unaffected by the relief fund, and overall profit margin (net income from all sources divided by total revenue, net of contractual allowances), which was affected by the relief fund, were calculated to measure operating profitability and overall financial viability, respectively.^4,5^ The unweighted mean and 95% CIs for the 2 measures were calculated from 2016 to 2020. Because hospitals recorded the money they received from the relief fund as “other nonoperating income,” the share of other nonoperating income to total revenue was also examined. To assess heterogeneity across hospitals characteristics, the trends of operating margin and overall profit margin were presented by ownership type (nonprofit, for profit, government), geographic location (metropolitan, micropolitan, rural), number of admissions (using tertiles), and the interactions between ownership type and geographic location.

As a robustness check, the operating margin, overall profit margin, and share of other income for hospitals that began their fiscal years on July 1 were calculated during 2016 to 2020. As of February 2, 2022, 785 hospitals that had continuous observations during this period were available in the data set.

This cross-sectional study followed the Strengthening the Reporting of Observational Studies in Epidemiology (STROBE) reporting guideline. Two-sided *t* tests were used to compare means (significance level, *P* < .05). Statistical analysis was conducted using Stata, version 14 (StataCorp). The Johns Hopkins University institutional review board determined that this study did not qualify as human participants research; therefore, approval was not required.

## Results

**Figure 1.**
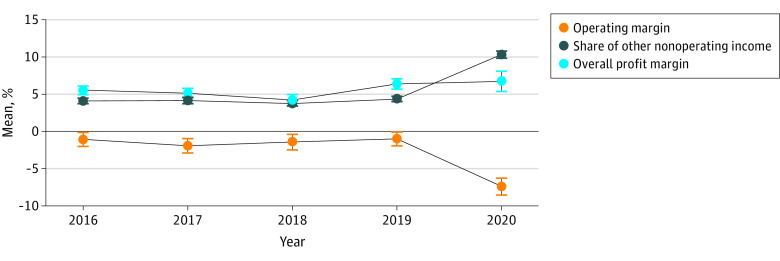
Trends of Hospital Operating Profitability and Financial Viability From 2016 to 2020 The sample contained 1378 hospitals that began their fiscal years on January 1, and 95% CIs are marked.

Among the 1378 hospitals, the mean operating margin declined from –1.0% (95% CI, –1.9% to –0.1%) in 2019 to –7.4% (95% CI, –8.5% to –6.3%) in 2020. The mean share of other nonoperating income grew from 4.4% (95% CI, 4% to 4.7%) in 2019 to 10.3% (95% CI, 9.9% to 10.8%) in 2020, and the mean overall profit margin in 2020 (6.7%; 95% CI, 5.4% to 8.1%) remained similar to that in prior years (Figure 1).

**Figure 2.**
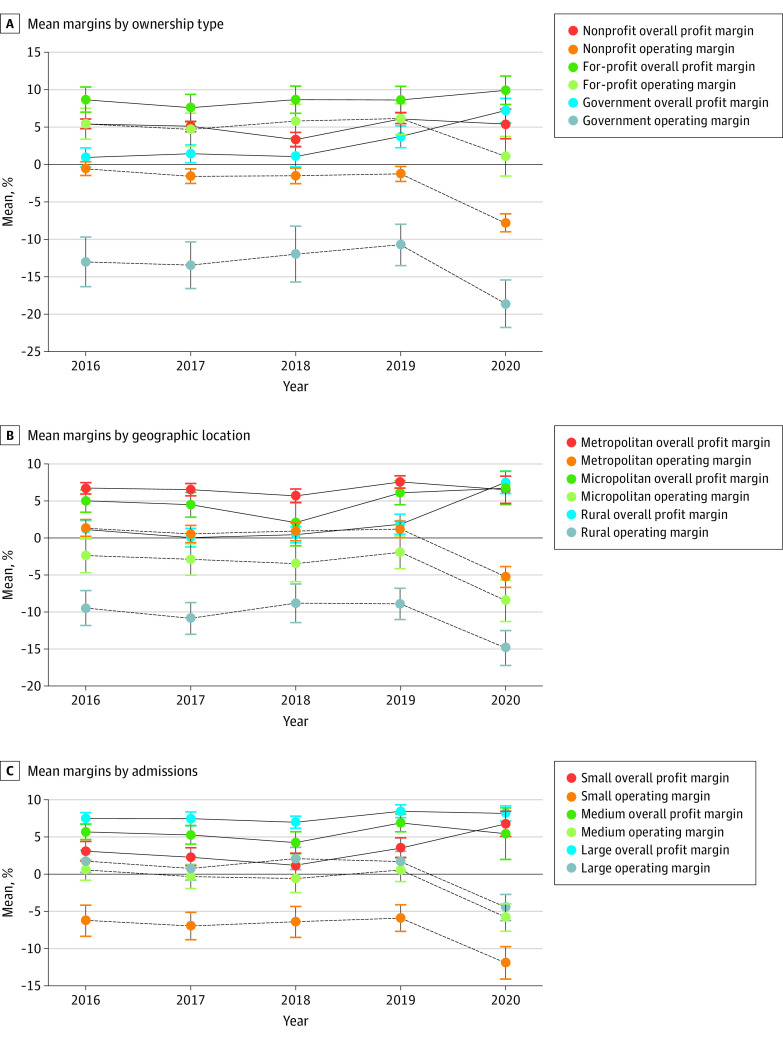
Trends of Hospital Financial Viability From 2016 to 2020 The sample contains 1378 hospitals that began their fiscal years on January 1, and 95% CIs are marked.

**Figure 3.**
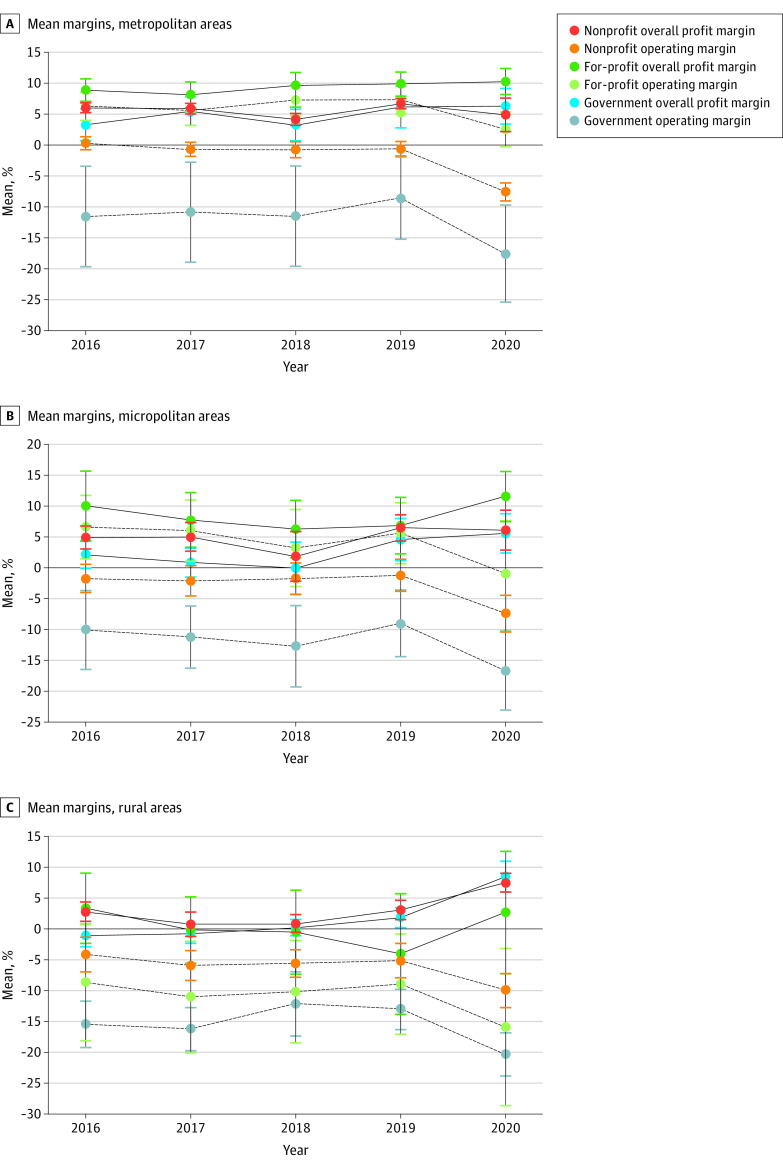
Trends of Hospital Financial Viability by the Interaction Between Ownership Type and Geographic Location From 2016 to 2020 The graphs illustrate a total of 934 (A), 212 (B), and 232 (C) hospitals that began their fiscal years on January 1. The 95% CIs are marked.

The mean operating margin declined, but the mean overall profit margin remained stable in 2020 for all ownership types, geographic locations, and size categories (Figure 2). In particular, government, rural, and smaller hospitals showed higher mean overall profit margins during 2020 than 2019 (2020: 7.2% [95% CI, 5.6% to 8.8%], 7.5% [95% CI, 6.0% to 9.0%], and 6.7% [95% CI, 5.1% to 8.3%], respectively; 2019: 3.7% [95% CI, 2.3% to 5.2%], 1.9% [95% CI, 0.5% to 3.2%], and 3.5% [95% CI, 2.1% to 4.8%], respectively). The interactions between ownership type and geographic location demonstrated the same pattern (Figure 3).

**Figure 4.**
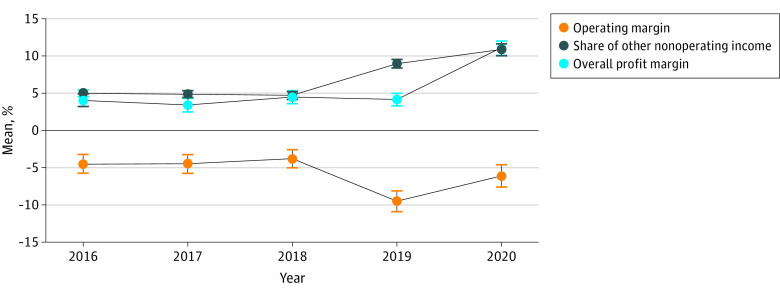
Sensitivity Analysis Results Examining Trends of Hospital Financial Viability From 2016 to 2020 The sample contains 785 hospitals that began their fiscal years on July 1, and 95% CIs are marked.

Unlike hospitals whose fiscal years began on January 1, hospitals whose fiscal years began on July 1 already entered the COVID-19 pandemic and started receiving COVID relief subsidies during the last 4 months of their fiscal year in 2019 (ie, March-June 2019). These hospitals experienced a sizable decline in operating margin during 2019 (mean, –9.5%; 95% CI, –10.9% to –8.1%) and 2020 (mean, –6.1%; 95% CI, –7.6% to –4.6%) (Figure 4). However, because they received relief funds during 2019 and 2020, their overall profit margin remained stable in 2019 (mean, 4.2%; 95% CI, 3.3% to 5.0%) and increased substantially in 2020 (mean, 11.1%; 95% CI, 10.1% to 12.0%).

## Discussion

This cross-sectional study found that in 2020, US hospitals experienced a sizeable reduction in operating margins. However, their overall profit margins remained similar to those in prior years, suggesting that the COVID-19 relief fund had effectively offset the financial losses of hospitals during the COVID-19 era. This was especially true for government, rural, and smaller hospitals that are typically more financially vulnerable. Compared with other hospitals, these hospitals received more relief fund compared with their scales of operations, as intended by some targeted funds allocations,^6^ which contributed to their higher overall profit margins in 2020 than in prior years.

### Limitations

The results of this study cannot be used to make quantitative inferences for all hospitals or individual hospitals. A hospital’s financial performance is associated with various factors, such as market characteristics, demographic characteristics of patients, and regulatory environment, which were not examined in this study. The results are also subject to potential measurement noise arising from reporting inaccuracies in administrative data. Despite these limitations, this study potentially facilitates understanding of the operational and overall financial implications of the COVID-19 pandemic.

## Conclusions

Although hospitals experienced a sizeable reduction in operating margins in 2020, their overall profit margins remained similar to those in prior years, suggesting that the COVID-19 relief fund effectively offset the financial losses for hospitals during the COVID-19 pandemic. Government, rural, and smaller hospitals, which were supported by some targeted fund allocations, generated higher overall profit margins during 2020 than in prior years.

## Notes

Supplement. **eTable 1.** Distribution of Medicare-certified General Acute Care Hospitals, by Fiscal Year Starting Month, 2019 vs. 2020 **eTable 2.** Sample Description
