# Supplementary material for: COVID-19 and Hospital Financial Viability in the US
Source: JAMA Health Forum. 2022 May 13;3(5):e221018. doi: 10.1001/jamahealthforum.2022.1018 (PMC9107033; doi:10.1001/jamahealthforum.2022.1018)
Supplement: Supplement. — eTable 1. Distribution of Medicare-certified General Acute Care Hospitals, by Fiscal Year Starting Month, 2019 vs. 2020 eTable 2. Sample Description [file jamahealthforum-e221018-s001.pdf]

Supplemental Online Content

Wang Y, Bai G, Anderson G. COVID-19 and hospital financial viability in the US. *JAMA Health Forum*. 2022;3(5):e221018. doi:10.1001/jamahealthforum.2022.1018

eTable 1. Distribution of Medicare-certified General Acute Care Hospitals, by Fiscal Year Starting Month, 2019 vs. 2020
eTable 2. Sample Description

This supplemental material has been provided by the authors to give readers additional information about their work.

© 2022 Wang Y et al. *JAMA Health Forum.*

eTable 1: Distribution of Medicare-certified General Acute Care Hospitals, by Fiscal Year Starting Month, 2019 vs. 2020 (data collected on February 2, 2022)

Fiscal year beginning	2019 # hospitals (proportion)	2020 # available hospitals
January 1	1,715 (39%)	1,695
February 1	27 (1%)	22
March 1	30 (1%)	30
April 1	106 (2%)	106
May 1	67 (2%)	64
June 1	74 (2%)	77
July 1	1,366 (31%)	964
August 1	33 (1%)	5
September 1	91 (2%)	2
October 1	790 (18%)	781
November 1	37 (1%)	41
December 1	24 (1%)	29
**Total**	**4,360 (100%)**	**3,816**

[a] Hospitals with Medicare Cost Reports available as of February 2, 2022.

© 2022 Wang Y et al. *JAMA Health Forum.*

eTable 2: Sample Description

Description	# hospital-year observation
Hospital-years with fiscal years starting on January 1, 2016-2020	8,541
-Hospital-years with missing financial performance measures	(315)
-Hospital-years with charge-to-cost ratio less than 0.2 or greater than 15.0	(11)
-Hospital-years with expense-to-revenue ratio less than 0.5 or greater than 3.0	(43)
-Hospital-years without five-year continuous observations for the same hospital	(1,282)
**Final sample**	**6,890**[a]

[a] The 6,890 hospital-year observations represent 1,378 unique hospitals, all of which have continuous observations during 2016-2020.

© 2022 Wang Y et al. *JAMA Health Forum.*
